# Prevalence and characteristics of adults with atherosclerotic cardiovascular disease, chronic kidney disease, and systemic inflammation in the USA

**DOI:** 10.1016/j.ahjo.2026.100772

**Published:** 2026-03-26

**Authors:** Michael G. Nanna, Lance A. Sloan, Ann Marie Navar, Mads D. Faurby, Lise Lotte N. Husemoen, Riyanka Paul, Gamze Tombak, Naveed Sattar

**Affiliations:** a Department of Internal Medicine, Section of Cardiovascular Medicine, Yale School of Medicine, Yale University, New Haven, CT, USA; b Texas Institute for Kidney and Endocrine Disorders, Lufkin, TX, USA; c Department of Internal Medicine, University of Texas Medical Branch, Galveston, TX, USA; d Division of Cardiology, Department of Internal Medicine, UT Southwestern Medical Center, Dallas, TX, USA; e Novo Nordisk Inc, Plainsboro, NJ, USA; f Novo Nordisk A/S, Søborg, Denmark; g Novo Nordisk Service Centre Pvt Ltd, Bangalore, India; h School of Cardiovascular & Metabolic Health, University of Glasgow, Glasgow, UK

**Keywords:** Atherosclerotic cardiovascular disease, Chronic kidney disease, Epidemiology, Prevalence, Inflammation

## Abstract

**Study objective:**

Systemic inflammation increases the risk of cardiovascular events and is often present in patients with atherosclerotic cardiovascular disease (ASCVD), particularly in those who also have chronic kidney disease (CKD). However, the prevalence of systemic inflammation in patients with ASCVD and CKD is not well characterized. Here, we estimate the prevalence of ASCVD with CKD and systemic inflammation in the US population and describe the characteristics of people with ASCVD, CKD stages 3–4, and systemic inflammation, who are at high risk for cardiorenal events.

**Design and setting:**

Cross-sectional study using data from the National Health and Nutrition Examination Survey (NHANES) 2015–2020 continuous cycles.

**Participants:**

Adults aged ≥ 20 years with ASCVD who had high-sensitivity C-reactive protein (hsCRP) measurements.

**Main outcome measures:**

Prevalence and characteristics of adults with systemic inflammation in groups with ASCVD, with ASCVD and CKD, and with ASCVD and CKD stages 3–4. Systemic inflammation was defined as hsCRP levels ≥ 2 mg/L.

**Results:**

Our study sample included 238,164,067 adults. In total, 8.5% had ASCVD, of whom 55.5% had systemic inflammation. The prevalence of systemic inflammation in individuals with ASCVD and CKD stages 3–4 (1.6% of the US population) was 59.1%, equating to 0.9% or > 2 million individuals in the general US adult population.

**Conclusions:**

In summary, over half of US adults with ASCVD and CKD stages 3–4 were estimated to have systemic inflammation. This group could benefit from weight loss strategies, lifestyle changes, and other approaches to improve cardiovascular outcomes.

## Highlights

• In this cross-sectional study of 2015–20 NHANES data, 8.5% of US adults had ASCVD.
• A total of 1.6% of US adults had ASCVD and CKD stages 3–4.
• Of those with ASCVD and CKD stages 3–4, 59.3% had systemic inflammation.
• Systemic inflammation is highly prevalent among groups with ASCVD and CKD.
• This suggests that systemic inflammation should be considered for risk assessment.

## 1 Introduction

Despite advances in the management of atherosclerotic cardiovascular disease (ASCVD) and improvements in cardiovascular (CV) outcomes, ASCVD remains a leading cause of morbidity and mortality in the United States and globally [1]. Inflammatory processes are involved in the pathogenesis of ASCVD, the development and progression of arterial plaques, and the occurrence of CV events [2], [3]. Importantly, even with optimal pharmacological and lifestyle interventions, systemic inflammation often remains in patients with ASCVD, putting them at risk of recurrent CV events [4], [5]. Chronic kidney disease (CKD) is also associated with systemic inflammation and with an increased risk of CV events and mortality [6], [7], [8], [9], [10]. Many patients with CKD have residual CV risk, defined as the risk of a recurrent CV event despite receiving the guideline-recommended standard of care [11], [12].

C-reactive protein (CRP) is a clinical biomarker of systemic inflammation. In clinical practice, systemic inflammation is quantified using high-sensitivity assays to detect low levels of CRP and is often defined as levels of high-sensitivity CRP (hsCRP) of ≥ 2 mg/L in the context of medical research and clinical trials [13], [14], [15], [16], [17]. hsCRP has been shown to be an independent predictor of CV events in several studies, including in patients at risk of CV events who are receiving statin therapy [18] or who are intolerant to statins [19].

Results from randomized controlled trials have provided proof of concept for therapies that target the innate immunity pathway that comprises the NLRP3 inflammasome, interleukin (IL)-1, and IL-6 as potential approaches for preventing and treating cardiovascular disease (CVD) and reducing systemic inflammation [17], [20], [21], [22]. In 2023, the US 10.13039/100009210Food and Drug Administration approved colchicine for the reduction of CV risk in adults with established ASCVD or with multiple risk factors for CVD, supported by findings from two phase 3 trials [21], [22], [23]. However, the use of colchicine in this population is contraindicated in patients with renal failure and it is advised to monitor patients with any degree of renal impairment for colchicine toxicity [23]. The CANTOS phase 3 CV outcomes trial showed that IL-1β inhibition with canakinumab significantly reduced CV event rates in patients with previous myocardial infarction and systemic inflammation at a median follow-up of 3.7 years [20]. In a sub-study of CANTOS, inflammatory risk appeared to be a potential factor in determining the risk of recurrent CV events in patients with ASCVD and impaired kidney function who were receiving statin therapy [12].

Despite the association of systemic inflammation with high CV risk, the precise epidemiologic burden of systemic inflammation and the associated risk among patients with ASCVD and CKD, as well as the demographics and clinical characteristics of this high-risk population, have not been well characterized. Here, we estimate the prevalence of ASCVD with CKD and systemic inflammation (as measured by hsCRP level) in the contemporary US population. We also describe the characteristics of people with ASCVD, CKD stages 3–4, and systemic inflammation, who are at high risk for cardiorenal events.

## 2 Methods

### 2.1 Data source

Individuals were identified in the National Health and Nutrition Examination Survey (NHANES) database, a nationally representative survey designed to be representative of the US population and used to assess the health and nutritional status of the noninstitutionalized population [24]. The survey examines a sample of approximately 5000 people each year, selected through a multi-stage area probability sampling design [24], [25]. Details of the survey methods, laboratory procedures, and data acquisition are provided on the NHANES website [26]. Ethical approval and informed consent were not required because the data were fully anonymized.

### 2.2 Study design and population

This cross-sectional study used data from the pre-COVID-19 pandemic, 2015–2020 NHANES cycles. Eligible adults (≥ 20 years old) were included if they had completed the NHANES Medical Conditions Questionnaire and had available data on serum creatinine, urine albumin to creatinine ratio (uACR), and hsCRP levels, in addition to age, sex, and race/ethnicity data.

### 2.3 Objectives

The primary objective was to estimate the prevalence of ASCVD with CKD and systemic inflammation in adults in the United States. Secondary objectives were: 1) to estimate the prevalence of systemic inflammation in adults with ASCVD without CKD and with CKD (any stage or stages 3–4); and 2) to describe the demographics, clinical characteristics, and self-reported hospital admissions of individuals with ASCVD and CKD stages 3–4 with systemic inflammation.

### 2.4 Definitions

ASCVD was self-reported in the NHANES Medical Conditions Questionnaire. Individuals with ASCVD were defined as those reporting at least one of the following conditions: previous coronary heart disease, angina/angina pectoris, heart attack, and/or stroke.

The estimated glomerular filtration rate (eGFR) was calculated using the 2021 Chronic Kidney Disease Epidemiology Collaboration (CKD-EPI) Eq. [27]. CKD stages were defined as per Kidney Disease: Improving Global Outcomes (KDIGO) international guidelines (KDIGO 2021 update of the 2012 guidelines) [28] based on eGFR category and albuminuria (absence of CKD: eGFR ≥ 60 mL/min/1.73 m^2^ and uACR < 30 mg/g; CKD stages 1–2: eGFR ≥ 60 mL/min/1.73 m^2^ and uACR ≥ 30 mg/g; CKD stage 3: eGFR 30 to < 60 mL/min/1.73 m^2^; CKD stage 4: eGFR 15 to < 30 mL/min/1.73 m^2^; CKD stages 3–4: eGFR 15 to < 60 mL/min/1.73 m^2^; and CKD stage 5 or end-stage renal disease: eGFR < 15 mL/min/1.73 m^2^).

Systemic inflammation was defined as hsCRP ≥ 2 mg/L; per NHANES documentation, CRP is measured using an immunoturbidimetric assay in a central laboratory [29].

### 2.5 Analyses

The population counts were derived from Centers for Disease Control and Prevention analytic guidelines [30] using population totals from the American Community Survey, which reflects the US population from 2015 and 2018. All outcomes were assessed after being weighted toward the US civilian noninstitutionalized resident population. Prevalence in the US population is reported as percent with 95% confidence intervals (CIs), which were estimated for the weighted results using Korn-Graubard CIs for weighted proportions [31].

For continuous variables, means and standard deviations (SDs) are presented; for categorical variables, numbers and percentages are shown.

Patient characteristics included self-reported characteristics (age, sex, race/ethnicity, and smoking status). Comorbidities (hypertension, previous myocardial infarction, previous stroke, heart failure, chronic obstructive pulmonary disease, type 2 diabetes, and prediabetes; all self-reported and/or based on laboratory measurements), and self-reported drug utilization (CV medications, antidiabetic medications, antihypertensive medications, lipid-lowering medications, and metabolic agents), including statins, β-adrenergic blocking agents, insulin, diuretics, angiotensin-converting enzyme (ACE) inhibitors, angiotensin receptor blockers, antiplatelet agents, and anticoagulants were also assessed. Data on healthcare resource utilization (overnight hospital admission within the past 12 months) were also analyzed.

## 3 Results

### 3.1 Study population

We identified 12,715 individuals in the NHANES 2015–2020 cycles aged 20 years or older with available data for the study (Supplementary Fig. 1). Weighting toward the general US population resulted in a population of 238,164,067; this group formed the cohort for this analysis.

### 3.2 Prevalence of ASCVD, CKD, and systemic inflammation

Within the weighted US population, an estimated 20,209,600 individuals (8.5%) had ASCVD, 6,743,019 individuals (2.8%) had ASCVD and CKD (stages 1–5), and 3,753,502 individuals (1.6%) had ASCVD and CKD stages 3–4 (Table 1).

**Table 1:** Prevalence estimates of ASCVD and ASCVD with CKD (stages 1–5, stages 1–2, stages 3–4, and stage 5) among the US population.

Group	Unweighted data (*N* = 12,715)	Weighted data (*N* = 238,164,067)	
	*n*	*n*	Prevalence, % (95% CI)
ASCVD	1331	20,209,600	8.49 (7.50–9.55)
ASCVD and CKD stages 1–5	553	6,743,019	2.83 (2.44–3.26)
ASCVD and CKD stages 1–2	226	2,797,718	1.17 (0.99–1.38)
ASCVD and CKD stages 3–4	310	3,753,502	1.58 (1.31–1.88)
ASCVD and CKD stage 5	17	191,800	0.08 (0.03–0.16)

Abbreviations: ASCVD, atherosclerotic cardiovascular disease; CKD, chronic kidney disease.

**Fig. 1:**
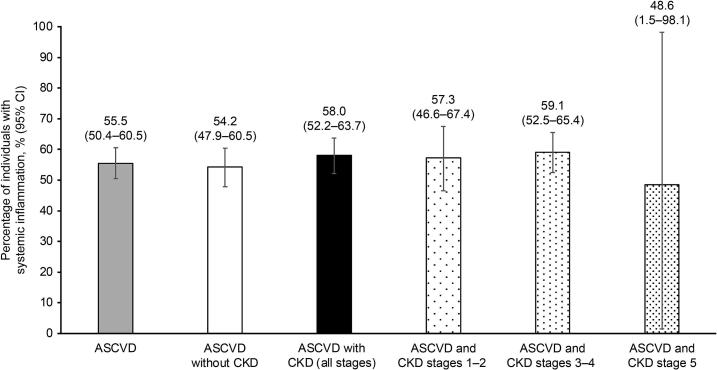
Prevalence estimates of systemic inflammation^a^ among individuals with ASCVD, individuals with ASCVD without CKD, and individuals with ASCVD and CKD (stages 1–5, stages 1–2, stages 3–4, and stage 5).^b^ Abbreviations: ASCVD, atherosclerotic cardiovascular disease; CI, confidence interval; CKD, chronic kidney disease; hsCRP, high-sensitivity C-reactive protein. ^a^ Defined as hsCRP ≥ 2 mg/L. ^b^ Presented as weighted data.

The estimated prevalence of systemic inflammation among the US population was more than 50% in the group with ASCVD, the group with ASCVD without CKD, and the group with ASCVD and CKD stages 1–5 (Fig. 1; Supplementary Table 1); the highest prevalence was observed in individuals with ASCVD and CKD stages 3–4 (59.1%).

### 3.3 Distribution of hsCRP levels in individuals with ASCVD and CKD (stages 1–5 and stages 3–4)

**Fig. 2:**
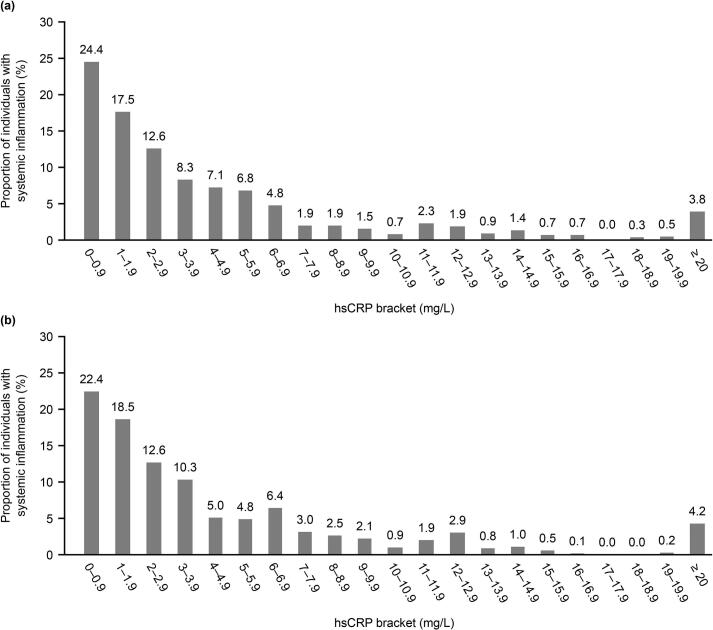
Distribution of hsCRP levels in individuals with ASCVD and CKD stages 1–5 (a) and in individuals with ASCVD and CKD stages 3–4 (b)^a^ (weighted data). Abbreviations: ASCVD, atherosclerotic cardiovascular disease; CKD, chronic kidney disease; CKD-EPI, Chronic Kidney Disease Epidemiology Collaboration; eGFR, estimated glomerular filtration rate; hsCRP, high-sensitivity C-reactive protein; KDIGO, Kidney Disease: Improving Global Outcomes. ^a^eGFR calculated using 2021 CKD-EPI equation and CKD stages defined as per KDIGO guidelines.

Among individuals with ASCVD and CKD (stages 1–5), 44.9% (*n* = 3,027,280) had hsCRP levels from 2 mg/L to < 10 mg/L, 13.1% (*n* = 886,027) had hsCRP levels ≥ 10 mg/L, and 3.8% (*n* = 259,091) had hsCRP levels ≥ 20 mg/L (Fig. 2a).

For individuals with ASCVD and CKD stages 3–4, 46.6% (*n* = 1,749,744) had hsCRP levels ≥ 2 mg/L and < 10 mg/L and 12.5% (*n* = 468,542) had hsCRP levels ≥ 10 mg/L (Fig. 2b); 4.2% of individuals (*n* = 157,285) with ASCVD and CKD stages 3–4 had hsCRP levels ≥ 20 mg/L.

In total, 2,218,285 individuals were estimated to have ASCVD with CKD stages 3–4 and systemic inflammation, which corresponds to 0.9% of the US adult population (*n* = 238,164,067) (Table 2).

**Table 2:** Prevalence estimates of systemic inflammation^a^ and ASCVD by presence or absence of CKD (stages 1–5, stages 3–4, and stage 5) among the US population.

Group	Unweighted data (*N* = 12,715)	Weighted data (*N* = 238,164,067)	
	*n*	*n*	Prevalence, % (95% CI)
ASCVD without CKD, with systemic inflammation	418	7,303,048	3.07 (2.54–3.66)
ASCVD, CKD stages 1–5 and systemic inflammation	337	3,913,306	1.64 (1.37–1.95)
ASCVD, CKD stages 1–2 and systemic inflammation	130	1,601,869	0.67 (0.53–0.84)
ASCVD, CKD stages 3–4 and systemic inflammation	194	2,218,285	0.93 (0.74–1.15)
ASCVD, CKD stage 5 and systemic inflammation	13	93,152	0.04 (0.02–0.06)

Abbreviations: ASCVD, atherosclerotic cardiovascular disease; CKD, chronic kidney disease; hsCRP, high-sensitivity C-reactive protein.

a Defined as hsCRP ≥ 2 mg/L.

### 3.4 Characteristics of individuals with ASCVD and CKD stages 3–4 and systemic inflammation

The weighted mean age was 72.4 years (SD, 8.3) for individuals with ASCVD, CKD stages 3–4, and systemic inflammation, versus 75.0 years (SD, 6.7) for individuals with ASCVD, CKD stages 3–4, without systemic inflammation. Of individuals with ASCVD, CKD stages 3–4, and systemic inflammation, 55.7% were women, 70.0% were White, and 17.2% were nonHispanic Black (Table 3). Most individuals (94.5%) were aged 60 years or older and approximately two-thirds (67.6%) were ever smokers. One-third of individuals (32.3%) were in obesity class II or III (body mass index [BMI] ≥ 35 kg/m^2^). High levels of cholesterol (≥ 190 mg/dL) and triglycerides (≥ 200 mg/dL) were observed in 31.0% and 23.8%, respectively, and 43.7% of individuals had moderate or severe albuminuria (uACR ≥ 30 mg/g).

**Table 3:** Characteristics, comorbidities, and healthcare resource utilization of individuals with ASCVD, CKD stages 3–4, with and without systemic inflammation.

Characteristic^a^	hsCRP ≥ 2 mg/L			hsCRP < 2 mg/L		
	*n*_unweighted_ (*N* = 194)	*n*_weighted_ (*N* = 2,052,913)	Weighted proportion	*n*_unweighted_ (*N* = 116)	*n*_weighted_ (*N* = 1,420,767)	Weighted proportion
**Demographic characteristics**						
Sex, women	85	1,143,174	55.7%	51	728,768	51.3%
Age categories						
20–< 30 years	0	0	0.0%	0	0	0.0%
30–< 40 years	1	16,964	0.8%	0	0	0.0%
40–< 50 years	2	12,055	0.6%	1	9521	0.7%
50–< 60 years	10	82,420	4.0%	4	51,370	3.6%
60–< 70 years	59	606,457	29.5%	17	127,342	9.0%
≥ 70 years	122	1,335,017	65.0%	94	1,232,534	86.8%
Race/ethnicity						
Mexican American	11	67,605	3.3%	8	46,070	3.2%
Other Hispanic	18	93,586	4.6%	6	34,778	2.4%
NonHispanic White	93	1,436,657	70.0%	61	1,042,180	73.4%
NonHispanic Black	61	353,157	17.2%	33	227,380	16.0%
NonHispanic Asian	4	25,509	1.2%	5	33,826	2.4%
Other or multiracial	7	76,399	3.7%	3	36,533	2.6%
Smoking status^b^ (*n* = 193/*n* = 116)^c^						
Ever smoked	126	1,382,993	67.6%	65	618,279	43.5%
BMI (*n* = 189/*n* = 113)^c^						
< 25 kg/m^2^	27	258,466	12.8%	26	267,490	19.3%
25–< 30 kg/m^2^	56	602,140	29.7%	49	615,156	44.5%
30–< 35 kg/m^2^	53	509,762	25.2%	27	324,967	23.5%
≥ 35 kg/m^2^	53	654,139	32.3%	11	176,076	12.7%
**Laboratory measurements**						
Total cholesterol						
< 70 mg/dL	0	0	0.0%	0	0	0.0%
70–< 100 mg/dL	6	43,260	2.1%	4	47,078	3.3%
100–< 130 mg/dL	21	221,451	10.8%	20	240,956	17.0%
130–< 160 mg/dL	53	567,275	27.6%	34	435,503	30.7%
160–< 190 mg/dL	59	584,787	28.5%	28	294,464	20.7%
≥ 190 mg/dL	55	636,140	31.0%	30	402,765	28.3%
Triglycerides (nonfasting, refrigerated serum)						
< 150 mg/dL	101	972,789	47.4%	69	790,426	55.6%
150–< 200 mg/dL	50	592,637	28.9%	30	400,994	28.2%
200–< 500 mg/dL	41	475,512	23.2%	17	229,347	16.1%
≥ 500 mg/dL	2	11,974	0.6%	0	0	0.0%
uACR						
< 30 mg/g	97	1,154,111	56.2%	69	909,544	64.0%
30–300 mg/g	61	612,726	29.8%	32	357,760	25.2%
> 300 mg/g	36	286,077	13.9%	15	153,463	10.8%
**CVD comorbidities**						
Heart failure^b^ (*n* = 194/*n* = 114)^c^	71	648,035	31.6%	28	299,333	21.3%
Previous MI^b^ (*n* = 193/*n* = 116)^c^	94	975,784	47.8%	52	611,040	43.0%
Previous stroke^b^ (*n* = 194/*n* = 115)^c^	87	781,097	38.0%	60	700,111	49.5%
**Other comorbidities**						
COPD, emphysema, chronic bronchitis (*n* = 192/*n* = 115)^c^	50	632,442	31.0%	21	262,351	18.5%
Hypertension^d^ (*n* = 172/*n* = 104)^c^	168	1,685,000	95.8%	102	1,203,555	96.9%
Overweight/obesity (*n* = 189/*n* = 113)^c^	162	1,766,041	87.2%	87	1,116,199	80.7%
Prediabetes^e^	50	489,045	23.8%	46	900,442	63.4%
Rheumatoid arthritis (*n* = 163/*n* = 104)^c^	24	214,499	12.6%	19	178,952	13.6%
Type 2 diabetes^f^	51	573,977	30.2%	30	393,190	28.5%
**Other characteristics**						
General health condition						
Excellent	4	40,976	2.0%	4	52,694	3.7%
Very good	25	276,643	13.5%	20	369,667	26.0%
Good	69	832,921	40.6%	43	478,709	33.7%
Fair	67	634,231	30.9%	39	444,041	31.3%
Poor	29	268,142	13.1%	10	75,655	5.3%
Medications (*n* = 193/*n* = 116)^c^						
ACE inhibitors	55	538,779	26.4%	33	407,309	28.7%
Anticoagulants	34	425,051	20.8%	17	229,105	16.1%
Antiplatelet agents	50	527,821	25.9%	26	391,512	27.6%
β-blockers	116	1,207,359	59.1%	63	851,191	59.9%
Diuretics	82	869,631	42.6%	35	450,226	31.7%
Statins	123	1,326,114	64.9%	72	933,840	65.7%
Glucose-lowering medication						
Biguanides	20	217,462	10.7%	18	223,669	15.7%
DPP-4is	11	119,121	5.8%	7	102,550	7.2%
Insulin	53	570,181	27.9%	17	150,370	10.6%
Sulfonylureas	20	241,053	11.8%	8	124,168	8.7%
HCRU						
Overnight hospital admission within the past 12 months	80	897,966	43.7%	33	431,476	30.4%
Number of times received healthcare within the past 12 months (*n* = 193/*n* = 116)^c^						
None	6	39,378	1.9%	4	40,585	2.9%
1	12	116,116	5.7%	6	56,010	3.9%
2–3	49	450,731	22.0%	21	228,451	16.1%
4–5	40	448,693	21.9%	31	365,868	25.8%
6–7	27	261,120	12.8%	16	166,136	11.7%
8–9	9	167,621	8.2%	8	132,424	9.3%
10–12	23	294,975	14.4%	18	296,061	20.8%
13–15	9	118,707	5.8%	6	35,736	2.5%
≥ 16	18	149,845	7.3%	6	99,497	7.0%

Abbreviations: ACE, angiotensin-converting enzyme; ASCVD, atherosclerotic cardiovascular disease; BMI, body mass index; CKD, chronic kidney disease; COPD, chronic obstructive pulmonary disease; CVD, cardiovascular disease; DBP, diastolic blood pressure; DPP-4i, dipeptidyl peptidase 4 inhibitor; HbA_1c_, glycated hemoglobin; HCRU, healthcare resource utilization; hsCRP, high-sensitivity C-reactive protein; MI, myocardial infarction; SBP, systolic blood pressure; uACR, urine albumin to creatinine ratio.

a Percentages are proportions of individuals among population with nonmissing data.

b Self-reported.

c For variables with missing data, the number of individuals with available data are shown in brackets (number of individuals with available data for groups with/without systemic inflammation).

d Self-reported or SBP ≥ 130 mm Hg or DBP ≥ 80 mm Hg.

e Self-reported or HbA_1c_ ≥ 5.7% and < 6.5%.

f HbA_1c_ ≥ 6.5% or self-reported; for identification of diabetes type, a treatment-based algorithm was used.

This population was characterized by a high comorbidity burden. A large proportion had hypertension (95.8%) and a history of CVD (previous myocardial infarction, 47.8%; previous stroke, 38.0%; and heart failure, 31.6%). The most frequently reported medications among individuals with ASCVD, CKD stages 3–4, and systemic inflammation were statins (64.9%), β-blockers (59.1%), diuretics (42.6%), insulin (27.9%), ACE inhibitors (26.4%), and antiplatelet agents (25.9%); other medications were reported by less than 25% of the population. Overnight hospital admission in the past year was reported by 43.7% of this population.

Characteristics, comorbidities, and healthcare resource utilization were similar when systemic inflammation was defined as hsCRP 2–10 mg/L, thereby excluding patients with extreme hsCRP values that could indicate acute inflammation (Supplementary Table 2).

## 4 Discussion

We aimed to determine the prevalence of individuals with ASCVD, CKD, and systemic inflammation (defined as hsCRP ≥ 2 mg/L) among the US population using NHANES data. We found that systemic inflammation was highly prevalent among groups at risk of CV events. The prevalence of systemic inflammation increased in the presence of CKD; prevalence was highest among people with ASCVD and CKD stages 3–4, of whom approximately 60% had systemic inflammation. Approximately 0.9% of the total adult population aged 20 years or older in the United States is estimated to have ASCVD, CKD stages 3–4, and systemic inflammation, representing approximately 2 million adults who have the potential to benefit from further targeted CV risk reduction efforts.

The prevalence estimates of systemic inflammation presented here are consistent with the results of previous studies assessing the prevalence of systemic inflammation in groups with a history of CVD. In a US retrospective study, 61% of adults with previous myocardial infarction identified from NHANES 1999–2010 had systemic inflammation (hsCRP levels ≥ 2 mg/L) [32], and, in a retrospective study from Germany, 43% of patients with coronary heart disease admitted to a general cardiology unit had hsCRP ≥ 2 mg/L [33]. The reported prevalence of systemic inflammation among people at a lower risk of CV events was generally lower. For example, in a 2018 study including women aged 18–40 years identified from the BioCycle study, the EAGeR trial, and NHANES, hsCRP levels from ≥ 2 mg/L to < 10 mg/L were present in 20–40% of participants. Notably, BMI and waist circumference were independently associated with higher hsCRP in all three cohorts [34]. Assessment of hsCRP levels is an important tool to help further refine risk assessment in patients with or who are at risk of ASCVD [35]. The American College of Cardiology/American Heart Association guideline on the primary prevention of CVD mention hsCRP as a possible risk enhancer in patients at intermediate risk [1], and the 2024 European Society for Cardiology guidelines for the management of chronic coronary syndromes now include a recommendation to consider hsCRP along with other biomarkers to refine risk stratification [36]. However, hsCRP assessment is rarely applied in the clinical setting.

Our results are clinically relevant because there is a significant need for further risk reduction in this population, given the risk of recurrent CV events despite guideline-directed treatment [18]. Notably, although US guidelines recommend that people with ASCVD receive statin treatment [1], only two-thirds of individuals with ASCVD, CKD stages 3–4, and systemic inflammation were receiving this treatment. Furthermore, despite the evidence showing a beneficial effect for ACE inhibitors in CKD [37], nearly half of the eligible population in our study were not receiving this medication. This gap in clinical care is consistent with other studies showing underutilization of preventive therapies in people with ASCVD [38], [39], [40].

It is increasingly considered that therapeutic management of individuals at risk of CV events should not only include strategies to reduce residual risk related to cholesterol, for example, via the use of statins or other lipid-lowering therapies, but also incorporate approaches to reduce the inflammatory risk in individuals for whom hsCRP remains at ≥ 2 mg/L despite optimal treatment [35]. Given that the prevalence of systemic inflammation was highest among people with ASCVD and CKD stages 3–4 in our study, targeting this specific group of patients may result in the highest absolute benefit.

Using NHANES data, which include comprehensive test results and measurements, we were able to produce reliable estimates for the contemporary prevalence of ASCVD, CKD, and systemic inflammation in a population representative of the general US population. The limitations of our study arose primarily from the data source. Owing to the extrapolation and weighting performed, our results depend on the representativeness of the NHANES data from 2015 to 2020 for the current US population. Data were available for hsCRP levels but not for other biomarkers of inflammation. Similarly, there is a lack of longitudinal measures of hsCRP in this database. The prevalence of ASCVD may be inaccurate, because ASCVD was self-reported and because peripheral artery disease or revascularization procedures were not included in the NHANES Medical Conditions Questionnaire. In addition, the study population may not be representative of people with later stages of CKD: only 0.1% of individuals had ASCVD and CKD stage 5 or end-stage renal disease (eGFR < 15 mL/min/1.73 m^2^), which is lower than the previously reported prevalence of CKD stage 5 in the US adult population (0.2% in 2003) [41]. This cross-sectional analysis used data from 2015 to 2020; the treatment landscape has changed since then, and medical therapies that may affect hsCRP levels have become standard of care. Further research is warranted to assess the impact of these newer therapies on the prevalence of systemic inflammation.

## 5 Conclusions

In this analysis of US NHANES data, systemic inflammation was common among people with ASCVD and was even more prevalent in people with ASCVD and CKD. Approximately 0.9% of the total adult population aged 20 years or older in the United States is estimated to have ASCVD, CKD stages 3–4, and systemic inflammation. This population, which is characterized by a high BMI and comorbidity burden, comprises an estimated 2 million adults who are likely to benefit from further targeted CV risk reduction efforts, in addition to current guideline-recommended medical therapy. Together with lifestyle changes, such as smoking cessation and sustained weight loss, targeted CV risk reduction has the potential to reduce CV events in these high-risk populations.

## CRediT authorship contribution statement

**Michael G. Nanna:** Writing – review & editing, Methodology, Conceptualization. **Lance A. Sloan:** Writing – review & editing, Conceptualization. **Ann Marie Navar:** Writing – review & editing, Methodology, Conceptualization. **Mads D. Faurby:** Writing – review & editing, Conceptualization. **Lise Lotte N. Husemoen:** Writing – review & editing, Conceptualization. **Riyanka Paul:** Writing – review & editing, Data curation, Conceptualization, Methodology. **Gamze Tombak:** Writing – review & editing, Conceptualization. **Naveed Sattar:** Writing – review & editing, Conceptualization.

## Ethical statement

• Ethical approval and informed consent were not required because the data were fully anonymized.
• The work described has not been published previously except in the form of an abstract and is not under consideration for publication elsewhere.
• The article's publication is approved by all authors.
• All funding sources have been acknowledged.

## Sources of funding

This study was funded by 10.13039/501100004191Novo Nordisk A/S.

## Declaration of competing interest

Dr. Nanna reports unrelated research funding from the American College of Cardiology (George F and Ann Harris Bellows Foundation), the Patient-Centered Outcomes Research Institute, the Yale Claude D. Pepper Older Americans Independence Center (P30AG021342), and the National Institute on Aging/National Institutes of Health (K76AG088428). Dr. Nanna also reports personal fees from HeartFlow, Novo Nordisk, and Merck. Dr. Sloan has served as a clinical investigator, consultant, and/or speaker for Abbott, Amgen, AstraZeneca, Bayer, Boehringer Ingelheim, Corcept, Eli Lilly, GlaxoSmithKline, Janssen, Merck, Novo Nordisk, Pfizer, and Sanofi Aventis. Dr. Navar reports funding for research to her institution from Amgen, Esperion, and Janssen, and honoraria and consulting fees from Amgen, Arrowhead, AstraZeneca, Bayer, Eli Lilly, Esperion, Janssen, Merck, New Amsterdam, Novartis, Novo Nordisk, Pfizer, Roche, and Silence Therapeutics. Mr. Faurby is an employee of Novo Nordisk Inc. Dr. Husemoen and Dr. Tombak are employees of Novo Nordisk A/S. Miss Paul is an employee of Novo Nordisk Service Centre India Pvt. Ltd., which is part of Novo Nordisk A/S. Mr. Faurby, Dr. Tombak, Dr. Husemoen, and Miss Paul are also shareholders of Novo Nordisk A/S. Dr. Sattar has served as a consultant and/or speaker for Abbott Diagnostics, Amgen, AstraZeneca, Boehringer Ingelheim, Eli Lilly, Hanmi, Novartis, Novo Nordisk, Pfizer, Roche Diagnostics, and Sanofi; he has received grant funding, paid to his university, from AstraZeneca, Boehringer Ingelheim, Novartis, and Roche Diagnostics.

## Data Availability

The data utilized in this study were derived from the National Health and Nutrition Examination Survey (NHANES) and are publicly accessible through the Centers for Disease Control and Prevention (CDC) website: https://www.cdc.gov/nchs/nhanes/
