# Supplementary material for: Prevalence and characteristics of adults with atherosclerotic cardiovascular disease, chronic kidney disease, and systemic inflammation in the USA
Source: Am Heart J Plus. 2026 Mar 26;65:100772. doi: 10.1016/j.ahjo.2026.100772 (PMC13066804; doi:10.1016/j.ahjo.2026.100772)
Supplement: Supplementary file 1 — Supplementary material [file mmc1.pdf]

Supplementary material

Prevalence and characteristics of adults with atherosclerotic cardiovascular disease, chronic kidney disease, and systemic inflammation in the USA

Authors:

Michael G. Nanna, MD, MHS[a,*], Lance A. Sloan, MD[b,c], Ann Marie Navar, MD, PhD[d], Mads D. Faurby, MSc[e], Lise Lotte N. Husemoen, PhD[f], Riyanka Paul, MSc[g], Gamze Tombak, MD[f], Naveed Sattar, MD, PhD[h]

Affiliations:

[a]*Department of Internal Medicine, Section of Cardiovascular Medicine, Yale School of Medicine, Yale University, New Haven, CT, USA*

[b]*Texas Institute for Kidney and Endocrine Disorders, Lufkin, TX, USA*

[c]*Department of Internal Medicine, University of Texas Medical Branch, Galveston, TX, USA*

[d]*Division of Cardiology, Department of Internal Medicine, UT Southwestern Medical Center, Dallas, TX, USA*

[e]*Novo Nordisk Inc, Plainsboro, NJ, USA*

[f]*Novo Nordisk A/S, Søborg, Denmark*

[g]*Novo Nordisk Service Centre Pvt Ltd, Bangalore, India*

[h]*School of Cardiovascular & Metabolic Health, University of Glasgow, Glasgow, UK*

Contents

Supplementary Fig. 1. Cohort for this analysis (unweighted population). 2

Supplementary Table 1 Prevalence estimates of systemic inflammationa among individuals with ASCVD, individuals with ASCVD without CKD, and individuals with ASCVD and CKD (stages 1–5, stages 1–2, stages 3–4, and stage 5). 3

Supplementary Table 2 Characteristics, comorbidities, and healthcare resource utilization of individuals with ASCVD, CKD stages 3–4,[a] with and without systemic inflammation (defined as hsCRP 2–10 mg/L). 4

Supplementary Fig. 1. Cohort for this analysis (unweighted population).

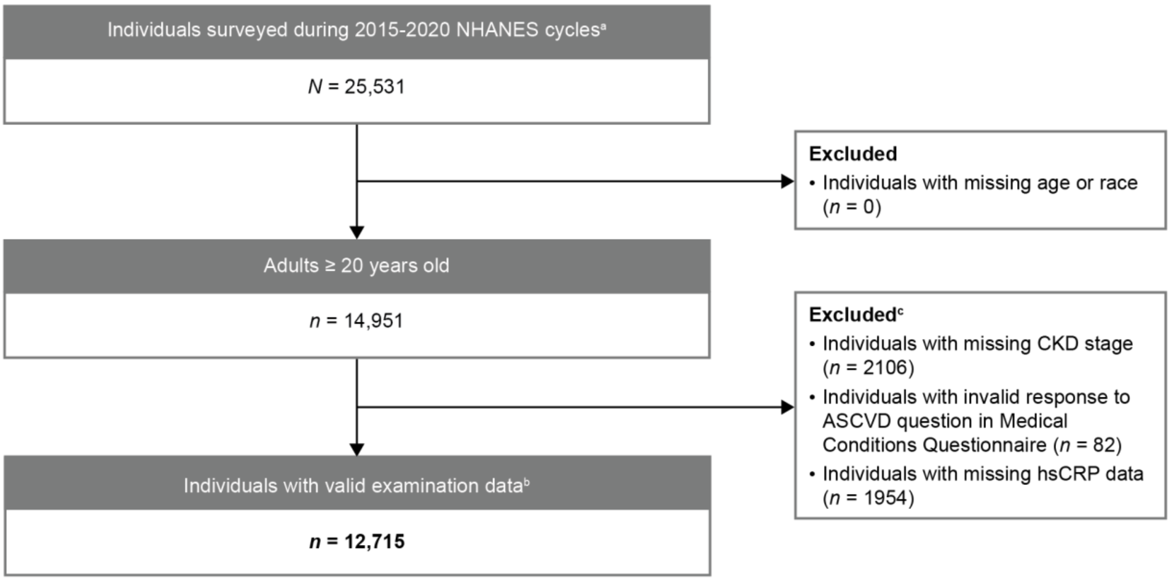

Abbreviations: CKD, chronic kidney disease; eGFR, estimated glomerular filtration rate; hsCRP, high-sensitivity C-reactive protein; NHANES, National Health and Nutrition Examination Survey; uACR, urine albumin to creatinine ratio.

[a] eGFR, uACR, hsCRP, and Medical Conditions Questionnaire data.

[b] In total, 9971 and 15,560 individuals were surveyed during 2015–2016 and 2017–2020 pre-pandemic data NHANES survey cycles, respectively.

[c] Exclusion criteria categories are overlapping; therefore, the number of individuals excluded does not match the total number of individuals in the categories.

Supplementary Table 1

Prevalence estimates of systemic inflammation[a] among individuals with ASCVD, individuals with ASCVD without CKD, and individuals with ASCVD and CKD (stages 1–5, stages 1–2, stages 3–4, and stage 5).

Group	Total population		With systemic inflammation		
				Weighted data	
	$n_{\text{unweighted}}$	n_{weighted}	$n_{unweighted}$	n	Prevalence, % (95% CI)
ASCVD	1331	20,209,600	755	11,216,355	55.50 (50.44–60.48)
ASCVD without CKD	778	13,466,581	418	7,303,048	54.23 (47.91–60.45)
ASCVD and CKD stages 1–5	553	6,743,019	337	3,913,306	58.03 (52.20–63.70)
ASCVD and CKD stages 1–2	226	2,797,718	130	1,601,869	57.26 (46.60–67.44)
ASCVD and CKD stages 3–4	310	3,753,502	194	2,218,285	59.10 (52.52–65.45)
ASCVD and CKD stage 5	17	191,800	13	93,152	48.57 (1.47–98.13)

Abbreviations: ASCVD, atherosclerotic cardiovascular disease; CKD, chronic kidney disease; hsCRP, high-sensitivity C-reactive protein.

[a] Defined as hsCRP ≥ 2 mg/L.

Supplementary Table 2

Characteristics, comorbidities, and healthcare resource utilization of individuals with ASCVD, CKD stages 3–4,[a] with and without systemic inflammation (defined as hsCRP 2–10 mg/L).

Characteristic[a]	hsCRP 2–10 mg/L			hsCRP < 2 mg/L		
	$n_{unweighted}$ ($N = 150$)	$n_{weighted}$ ($N = 1,619,301$)	Weighted proportion	$n_{unweighted}$ ($N = 116$)	$n_{weighted}$ ($N = 1,420,767$)	Weighted proportion
Demographic characteristics						
Sex, women	65	882,845	54.5%	51	728,768	51.3%
Age categories						
20–< 30 years	0	0	0.0%	0	0	0.0%
30–< 40 years	1	16,964	1.0%	0	0	0.0%
40–< 50 years	2	12,055	0.7%	1	9521	0.7%
50–< 60 years	6	51,770	3.2%	4	51,370	3.6%
60–< 70 years	48	528,512	32.6%	17	127,342	9.0%
≥ 70 years	93	1,010,000	62.4%	94	1,232,534	86.8%
Race/ethnicity						
Mexican American	5	29,189	1.8%	8	46,070	3.2%
Other Hispanic	15	77,279	4.8%	6	34,778	2.4%

NonHispanic White	74	1,154,950	71.3%	61	1,042,180	73.4%
NonHispanic Black	49	289,496	17.9%	33	227,380	16.0%
NonHispanic Asian	3	19,003	1.2%	5	33,826	2.4%
Other or multiracial	4	49,383	3.0%	3	36,533	2.6%
Smoking status[b]						
Ever smoked	98	1,118,816	69.1%	65	618,279	43.5%
BMI (n = 147/n = 113)[c]						
< 25 kg/m^2	21	225,904	14.1%	26	267,490	19.3%
25–< 30 kg/m^2	44	449,381	28.1%	49	615,156	44.5%
30–< 35 kg/m^2	39	352,286	22.0%	27	324,967	23.5%
≥ 35 kg/m^2	43	572,544	35.8%	11	176,076	12.7%
Laboratory measurements						
Total cholesterol						
< 70 mg/dL	0	0	0.0%	0	0	0.0%
70–< 100 mg/dL	6	43,260	2.7%	4	47,078	3.3%
100–< 130 mg/dL	15	172,352	10.6%	20	240,956	17.0%
130–< 160 mg/dL	44	498,522	30.8%	34	435,503	30.7%
160–< 190 mg/dL	44	442,018	27.3%	28	294,464	20.7%

≥ 190 mg/dL	41	463,149	28.6%	30	402,765	28.3%
Triglycerides (nonfasting, refrigerated serum)						
< 150 mg/dL	82	820,028	50.6%	69	790,426	55.6%
150–< 200 mg/dL	41	485,240	30.0%	30	400,994	28.2%
200–< 500 mg/dL	26	309,760	19.1%	17	229,347	16.1%
≥ 500 mg/dL	1	4272	0.3%	0	0	0.0%
uACR						
< 30 mg/g	85	1,022,037	63.1%	69	909,544	64.0%
30–300 mg/g	42	418,727	25.9%	32	357,760	25.2%
> 300 mg/g	23	178,537	11.0%	15	153,463	10.8%
CVD comorbidities						
Heart failure[b] (*n* = 150/*n* = 114)[c]	53	507,765	31.4%	28	299,333	21.3%
Previous MI[b] (*n* = 149/*n* = 116)[c]	69	775,562	48.3%	52	611,040	43.0%
Previous stroke[b] (*n* =150/*n* = 115)[c]	63	583,847	36.1%	60	700,111	49.5%
Other comorbidities						
COPD, emphysema, chronic bronchitis (*n* = 149/*n* = 115)[c]	37	516,846	32.0%	21	262,351	18.5%
Hypertension[d] (*n* = 134/*n* = 104)[c]	131	1,353,524	95.4%	102	1,203,555	96.9%
Overweight/obesity (*n* = 147/*n* = 113)[c]	126	1,374,211	85.9%	87	1,116,199	80.7%

Prediabetes[e]	41	425,103	26.3%	46	900,442	63.4%
Rheumatoid arthritis (*n* = 128/*n* = 104)[c]	17	170,785	12.7%	19	178,952	13.6%
Type 2 diabetes[f]	36	450,431	29.8%	30	393,190	28.5%
Other characteristics						
General health condition						
Excellent	3	36,323	2.2%	4	52,694	3.7%
Very good	19	196,939	12.2%	20	369,667	26.0%
Good	56	678,456	41.9%	43	478,709	33.7%
Fair	54	515,805	31.9%	39	444,041	31.3%
Poor	18	191,778	11.8%	10	75,655	5.3%
Medications (*n* = 149/*n* = 116)[c]						
ACE inhibitors	40	412,233	25.6%	33	407,309	28.7%
Anticoagulants	25	303,296	18.9%	17	229,105	16.1%
Antiplatelet agents	37	402,815	25.0%	26	391,512	27.6%
β-blockers	87	964,299	60.0%	63	851,191	59.9%
Diuretics	60	639,165	39.7%	35	450,226	31.7%
Statins	99	1,127,175	70.1%	72	933,840	65.7%
Glucose-lowering medication						
Biguanides	15	171,341	10.7%	18	223,669	15.7%

DPP-4is	8	84,928	5.3%	7	102,550	7.2%
Insulin	37	386,198	24.0%	17	150,370	10.6%
Sulfonylureas	15	191,305	11.9%	8	124,168	8.7%
HCRU						
Overnight hospital admission within the past 12 months	57	698,781	43.2%	33	431,476	30.4%
Number of times received healthcare within the past 12 months (n = 149/n = 116)[c]						
None	6	39,378	2.4%	4	40,585	2.9%
1	8	57,301	3.6%	6	56,010	3.9%
2–3	41	401,689	24.9%	21	228,451	16.1%
4–5	34	337,628	20.9%	31	365,868	25.8%
6–7	21	207,546	12.9%	16	166,136	11.7%
8–9	6	143,150	8.9%	8	132,424	9.3%
10–12	16	238,355	14.8%	18	296,061	20.8%
13–15	6	83,629	5.2%	6	35,736	2.5%
≥ 16	11	104,898	6.5%	6	99,497	7.0%

[a] Percentages are proportions of individuals among population with nonmissing data.

[b] Self-reported.

[c] For variables with missing data, the number of individuals with available data are shown in brackets (number of individuals with available data for groups with/without systemic inflammation).

[d] Self-reported or SBP ≥ 130 mm Hg or DBP ≥ 80 mm Hg.

[e] Self-reported or HbA_{1c} ≥ 5.7% and < 6.5%.

[f] HbA_{1c} ≥ 6.5% or self-reported; for identification of diabetes type, a treatment-based algorithm was used.

ACE, angiotensin-converting enzyme; ASCVD, atherosclerotic cardiovascular disease; BMI, body mass index; CKD, chronic kidney disease; COPD, chronic obstructive pulmonary disease; CVD, cardiovascular disease; DBP, diastolic blood pressure; DPP-4i, dipeptidyl peptidase 4 inhibitor; HbA_{1c}, glycated hemoglobin; HCRU, healthcare resource utilization; hsCRP, high-sensitivity C-reactive protein; MI, myocardial infarction; SBP, systolic blood pressure; uACR, urine albumin to creatinine ratio.
